# Scalable nanohybrids of graphitic carbon nitride and layered NiCo hydroxide for high supercapacitive performance†

**DOI:** 10.1039/c9ra06068e

**Published:** 2019-10-18

**Authors:** Bebi Patil, Changyong Park, Heejoon Ahn

**Affiliations:** Institute of Nano Science and Technology, Hanyang University Seoul 04763 South Korea ahn@hanyang.ac.kr; Department of Organic and Nano Engineering, Hanyang University Seoul 04763 South Korea

## Abstract

The limited number of edge nitrogen atoms and low intrinsic electrical conductivity hinder the supercapacitive energy storage applications of the nitrogen-rich graphitic carbon nitride (g-C_3_N_4_). In this study, a novel graphitic carbon nitride/NiCo-layered double hydroxide (CNLDH), a two-dimensional nanohybrid, is prepared by a simple hydrothermal synthesis. The homogeneous interpolation of g-C_3_N_4_ nanosheets into NiCo LDH stacked nanosheets effectively increases the overall performances of the g-C_3_N_4_/NiCo LDH nanohybrid. The improved morphology of the nanohybrid electrode upon the addition of g-C_3_N_4_ to the NiCo LDH yields a specific capacity of 183.43 mA h g^−1^ in 6 M KOH at 1 A g^−1^, higher than those of bare g-C_3_N_4_ (20.89 mA h g^−1^) and NiCo LDH (95.92 mA h g^−1^) electrodes. The excellent supercapacitive performance of the CNLDH nanohybrid is complemented by its low internal resistance, excellent rate capability, and large cycling lifetime. Furthermore, the hybrid supercapacitor is assembled using CNLDH 0.1 as a positive electrode and activated carbon (AC) as a negative electrode. The hybrid supercapacitor device of CNLDH 0.1//AC shows the maximum specific capacity of 37.44 mA h g^−1^ at 1 A g^−1^ with remarkable energy density, power density and good cycling performance. This confirms that the CNLDH 0.1 nanohybrid is an excellent electrode material for supercapacitor applications.

## Abstract 2

A two dimensional CNLDH 0.1 nanohybrid supercapacitor electrode prepared by simple hydrothermal hybridization of g-C_3_N_4_ and NiCO LDH shows the maximum specific capacity of 183.43 mA h g^−1^ with remarkable electrochemical performance.

## Graphical abstract

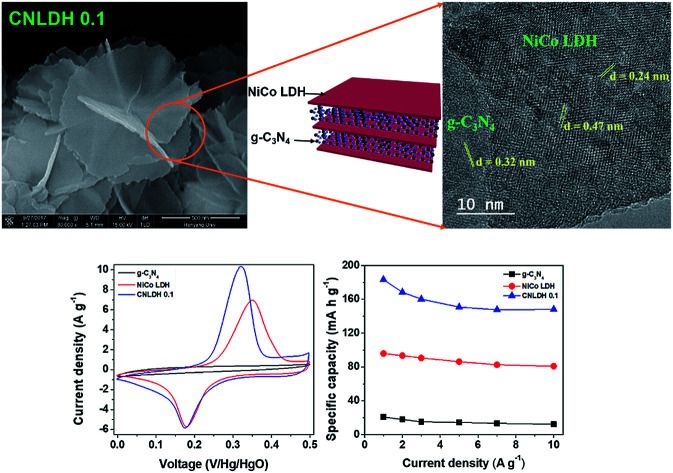

## Introduction

Energy storage technologies involving supercapacitors require high energy and power densities.^1^ To achieve this aim, extensive studies have been carried out on the development of new electrode materials that are superior to the existing class of metal oxides, carbon materials, conducting polymers, *etc.*^2–4^ Depending on the structures of the electrodes, the supercapacitors fundamentally operate based on two mechanisms, electrochemical double-layer capacitance and pseudo-capacitance, storing energy by charge accumulation at the electrode–electrolyte interface and by fast and reversible surface redox reactions, respectively.^5^ Regarding the material design, the development of hybrid nanostructures with different properties by combining various building blocks into newly designed structures has attracted large interest. The combination of two different nanostructures with distinct physical and chemical properties into a new hybrid with a unique structure can usually inherit cumulative advantages of the individual materials or even lead to the formation of a new heterostructured material with better properties. In recent years, the syntheses of different two-dimensional (2D) layered materials, such as graphene, hexagonal boron nitride, layered double hydroxides (LDHs), carbon nitride, MXenes, and transition metal dichalcogenides, have attracted considerable attention for the development of energy materials having unique properties.^6–9^ The 2D layered structures of these materials provide metallic or semiconducting properties.^10^ The 2D layered structures have advantages over bulk materials, such as the facilitated intercalation/deintercalation of ions and volume expansion,^11^ and thus have been employed in various applications, such as sensing,^12^ electrochemical energy storage and conversion,^13^ catalysis,^14^ and transistors.^15^ The increased interlayer distance in 2D layered materials is advantageous for energy storage as it can facilitate the ion transport and provide a better tolerance to the volume change, unlike in bulk materials.^16^ Additionally, the large surface area and number of active sites in 2D layered materials make them desirable for use as energy storage devices.

Despite the extensive studies on 2D layered materials, their functionality should be improved. A novel approach for this purpose is the development of heterostructures of organic–inorganic 2D layered materials. This approach can pave the way for the development of novel electrodes with increased electrochemical performances by combining the advantages of the individual building blocks while eliminating their shortcomings. To demonstrate our concept, we hybridize 2D graphitic carbon nitride (g-C_3_N_4_) and LDH of nickel–cobalt (NiCo). g-C_3_N_4_, a nanocarbon material consisting of two earth-abundant elements (carbon and nitrogen), is a novel 2D layered organic semiconductor^17^ which has attracted increasing interest owing to its layered structure, simple synthesis, and low synthesis cost. The LDHs, such as the NiCo LDHs, are a class of 2D inorganic layered matrices consisting of positively charged layers, with anions and water molecules intercalated in the interlayer region. The 2D NiCo LDH provides a short ion diffusion path, abundant active sites, and different valence states, resulting in an increased electrochemical performance.^18,19^ However, the limited electroactive sites, low conductivity, low cycling stability of the NiCo LDH, and low ion transport capability of g-C_3_N_4_ hinder the applications of both materials. To overcome the disadvantages of these materials, we develop integrated heterostructures of g-C_3_N_4_ with NiCo LDH by successfully coupling them. This concept elucidates the specific challenges that need to be addressed for the introduction of 2D heterostructured electrodes into next-generation energy storage devices.

## Experimental methods

### Materials

Analytically pure urea, nitric acid (HNO_3_), absolute ethanol (99%), nickel nitrate (Ni(NO_3_)_2_·6H_2_O), cobalt nitrate (Co(NO_3_)_2_·6H_2_O), and potassium hydroxide (KOH) were purchased from Sigma-Aldrich. None of the materials used in the experiments were further purified, unless otherwise stated.

### Synthesis

#### g-C_3_N_4_

g-C_3_N_4_ was fabricated by the simple pyrolysis of urea.^20^ The urea precursor (5 g) was kept in an alumina crucible with a cover. The crucible was heated for 2 h at 80 °C to remove moisture and obtain an oxygen-free product. The crucible was then heated at 550 °C in the ambient air for 3 h at a rate of 5 °C min^−1^. The resulting product was washed with 0.1 M of nitric acid and distilled water several times and dried at 80 °C for 24 h. A pale-yellow g-C_3_N_4_ powder was obtained after ultrasonication for 2 h.

#### NiCo LDH

The NiCo LDH powder was synthesized by a hydrothermal method. In the typical synthesis, 0.582 g of Ni(NO_3_)_2_·6H_2_O and 0.874 g of Co(NO_3_)_2_·6H_2_O were dissolved in 70% ethanol. The prepared solution was transferred in a 200 mL Teflon autoclave and heated at 160 °C for 3 h. During the cooling of the reaction mixture to room temperature, the resultant solution was washed with copious amounts of water and ethanol. The washed powder was kept in the vacuum oven at 60 °C for 12 h. Different nanostructures of NiCo LDH were synthesized by varying the synthesis time (6, 8, and 12 h).

#### g-C_3_N_4_–NiCo LDH (CNLDH) heterostructures

**Fig. 1:**
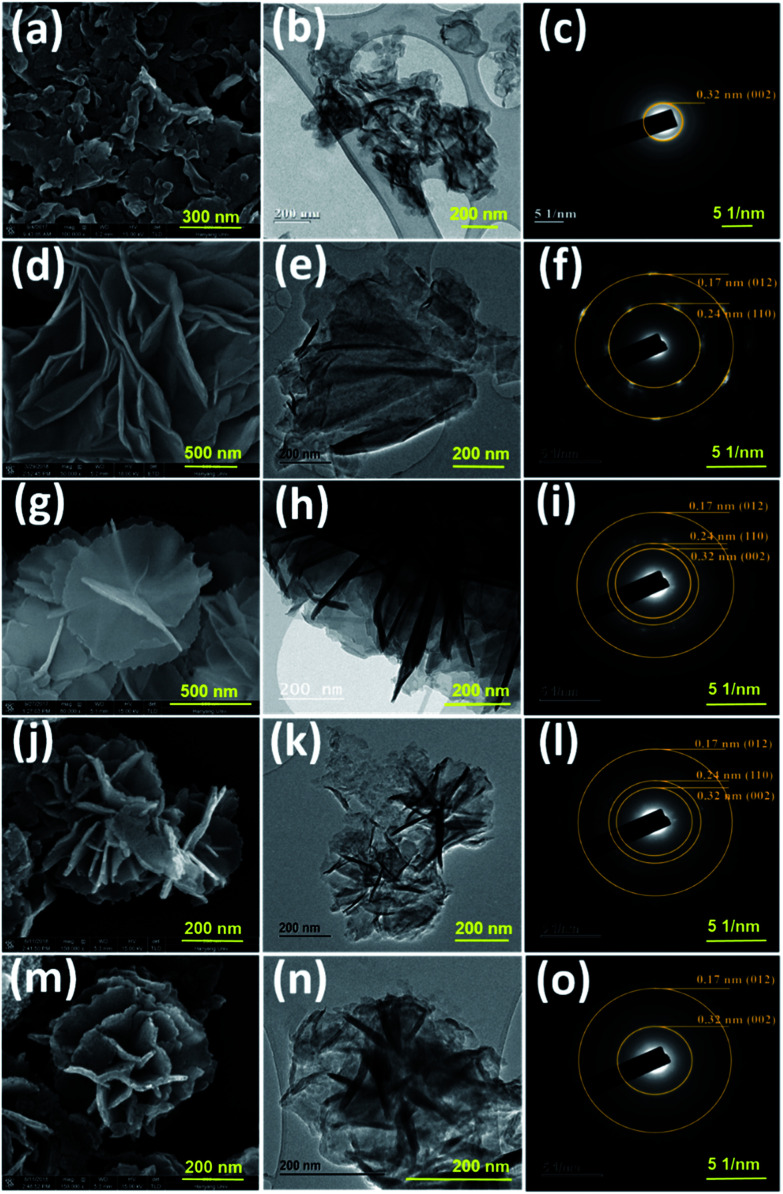
SEM and TEM images and selected-area electron diffraction patterns of (a–c) g-C_3_N_4_, (d–f) NiCo LDH, (g–i) CNLDH 0.1, (j–l) CNLDH 0.3, and (m–o) CNLDH 0.5, respectively.

The optimized NiCo LDH sample was further used for the preparation of CNLDH heterostructures. The CNLDH heterostructures were prepared by adding 0.1, 0.3, and 0.5 g of the as-prepared g-C_3_N_4_ to the Ni and Co precursors and carrying out the synthesis under the same set of conditions. The obtained products are denoted as CNLDH 0.1, 0.3, and 0.5, respectively. Fig. 1 illustrates the hybridization of g-C_3_N_4_ and NiCo LDH, carried out to form the layer-on-layer assembly of CNLDH heterostructure.

### Characterization

The morphologies and microstructures of the as-obtained products were characterized by field-emission scanning electron microscopy (FESEM, JEOL 7800), transmission electron microscopy (TEM, 2100F), and high-angle annular-dark-field scanning transmission electron microscopy (HAADF-STEM, JEM-ARM 200F). X-ray diffraction (XRD; MiniFlex 600) patterns were recorded using Cu K_α_ radiation (*λ* = 0.15406 nm). The specific surface area was calculated using the Brunauer–Emmett–Teller (BET) method, while the pore size distribution was obtained using the desorption branch data by the Barrett–Joyner–Halenda (BJH) method. Fourier-transform infrared (FTIR) spectroscopy was performed using a Nanofinder 3.0 Raman spectrometer at an excitation wavelength of 488 nm. X-ray photoelectron spectroscopy (XPS) was performed using a Kratos-Axis spectrometer with monochromatic Al K_α_ (1486.7 eV) X-ray radiation (15 kV, 10 mA). Electrochemical measurements of the g-C_3_N_4_, NiCo LDH, and CNLDH heterostructures were carried out in a three-electrode system, in a 6 M KOH aqueous solution, where Hg/HgO and Pt coil were used as the reference and counter electrodes, respectively. For the fabrication of the working electrode, the active material were mixed with poly (vinylidene fluoride) (PVDF, M. W. 534 000, Sigma-Aldrich) and carbon black (Super P, TIMCAL Graphite & Carbon) with a weight ratio of 8 : 1 : 1 in *N*-methyl-2-pyrrolidone (NMP) to form a slurry. The slurry was coated onto a carbon paper current collector (1 × 1 cm^2^) using a spatula. The prepared electrodes were dried at 110 °C for 12 h under vacuum. The same process followed for the preparation of activated carbon (AC) as a negative electrode for hybrid supercapacitor. The total mass of CNLDH 0.1 and AC was about 3.5 mg cm^−2^. Cyclic voltammetry (CV) and galvanostatic charge–discharge (GCD) measurements were carried out using an electrochemical workstation (ZIVE SP1, WonA Tech) in the range of 0 to +0.5 V/Hg/HgO for half-cell test and 0 to +1.2 V for hybrid supercapacitor at various scanning rates and current densities. Prior to the CV and GCD measurements, the electrodes were activated by cycling the operation voltage for 25 cycles at 50 mV s^−1^. The ion kinetics within the electrode material were investigated by electrochemical impedance spectroscopy (EIS) in a frequency range of 0.01 to 10^5^ Hz at the open-circuit potential. The specific capacity (*C*_sp_, mA h g^−1^), energy density (*E*, W h kg^−1^) and power density (*P*, W kg^−1^) were calculated using the GCD curve,*C*_sp_ = *I*Δ*t*/*m*,*E* = *I*Δ*tV*/*m*,and*P* = *E*/Δ*t*where *I* (mA), *t* (h), *V* (V) and *m* (g) are the discharging current, time, voltage and mass of active material, respectively.

## Results and discussion

### SEM

**Scheme 1:**
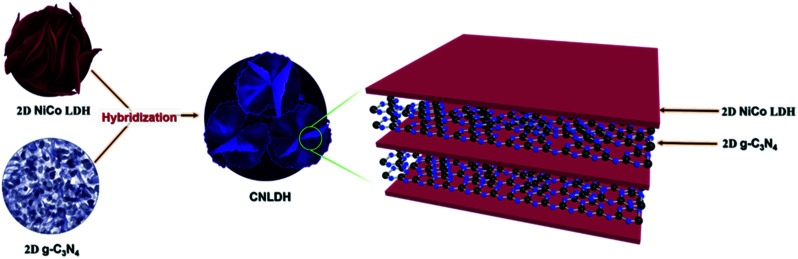
Schematic of the fabrication of the CNLDH nanohybrid.

The morphologies of the as-prepared nanostructures were characterized by FESEM and TEM. The SEM images of the NiCo LDHs in Fig. S1† reveal the separation of the LDH nanosheets with the increase in hydrothermal synthesis time. Typically, collective nanosheets with a thickness of ∼90 nm are formed by the synthesis for 3 h, whereas vertically aligned nanosheets having a thickness of ∼30 nm are obtained by the synthesis for 12 h (Fig. S1†). These thin nanosheets are densely interconnected with each other forming a highly porous network (Scheme 1).

The nanoporous structure could be beneficial to increase the electrochemical performance of the LDH. Therefore, the NiCo LDH obtained with the synthesis time of 12 h was considered ideal for the further preparation of CNLDH nanohybrids. The morphologies of the g-C_3_N_4_, NiCo LDH, CNLDH 0.1, CNLDH 0.3, and CNLDH 0.5 nanohybrids are presented in Fig. 1. The SEM image in Fig. 1a shows the lamellar crumpled silk-like structure of g-C_3_N_4_.^21^ As shown in Fig. 1b and c, the lamellar structure of g-C_3_N_4_ exhibits a lattice spacing of 0.32 nm corresponding to the (002) reflection of the graphitic structure of carbon nitride, which corroborates the XRD results. Fig. 1d–f shows the hydrothermally synthesized polycrystalline thin sheets of NiCo hydroxide. Notably, upon the introduction of the as-prepared g-C_3_N_4_ nanosheets in the hydrothermal autoclave, the CNLDH hybrid nanosheets could be synthesized in the form of “sheet-on-sheet” structure. In CNLDH 0.1 (Fig. 1g–i), the NiCo LDH and g-C_3_N_4_ are well intercalated. The g-C_3_N_4_ can effectively reduce the dimensions of the nanosheets by forming ultrathin nanolayers (CNLDH 0.1; Fig. 1g and h). Such structure could provide excellent electron-diffusion paths facilitating the diffusion and migration of electrolyte ions within the electrode materials and enhancing the electrochemical behavior of the nanohybrid.^22^ The morphological analyses suggest that with the increase in content of g-C_3_N_4_, LDH nanoflowers seem to grow on g-C_3_N_4_ nanosheets at the expense of the crystallinity (Fig. 1j–o). Such behavior has been also reported for an LDH/graphene oxide composite having a layer-on-layer structure.^23^

### HAADF STEM

**Fig. 2:**
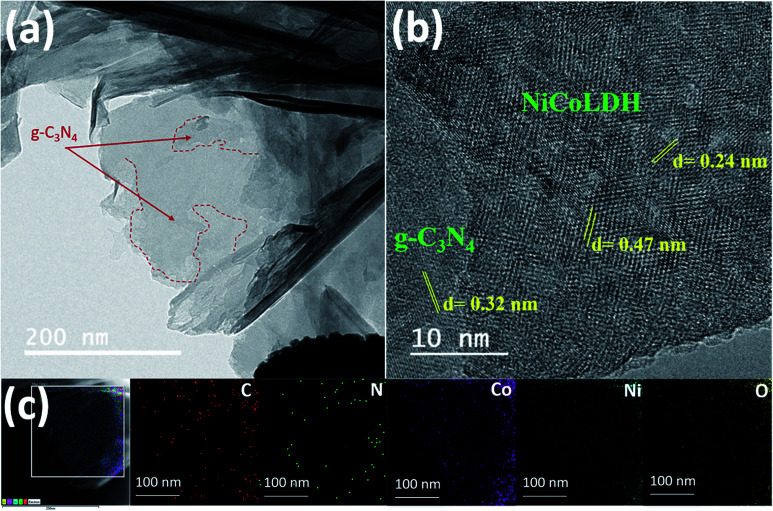
(a) TEM and (b) HAADF-STEM images and (c) elemental maps of CNLDH 0.1.

CNLDH 0.1 was investigated by HAADF-STEM. The elemental maps (Fig. 2c) show the uniform distributions of C and N elements. The TEM image (Fig. 2a) of CNLDH 0.1 shows that ultrathin NiCo LDH nanosheets densely cover the surface of g-C_3_N_4_ (outlined by red dashed lines). The firmly immobilized NiCo LDH nanosheets obtained during the TEM specimen preparation remained unseparated for days, indicative of a strong interaction between g-C_3_N_4_ and NiCo LDH. The high-resolution TEM image in Fig. 2b shows the calculated lattice spacings of the nanosheets of approximately 0.24 and 0.47 nm, corresponding to the (110) and (006) planes of the NiCo LDH, respectively, and that of 0.32 nm, corresponding to the (002) planes of g-C_3_N_4_. Energy-dispersive X-ray spectroscopy (EDS) mapping was carried out to determine the compositional and elemental distributions; the results are shown in Fig. 2c. The uniform distributions of the characteristic elements C, N, Ni, Co, and O in the EDS maps demonstrate the presence of g-C_3_N_4_ nanosheets between the layers of the NiCo LDH. Such unique 2D heterostructure can provide more electrochemically active sites for the exposure to the electrolyte, favoring the ion transfer and diffusion and accelerating the surface redox reaction.^24^

### XRD

The sharp peaks in the XRD patterns of the NiCo LDHs obtained using different hydrothermal synthesis times (Fig. S2†) indicate the considerable crystallinities of the as-synthesized NiCo-based hydroxide samples, which is attributed to the hydrothermal reaction environment that is carefully engineered for crystal growth. Moreover, the absence of other peaks related to side reactions and impurities reflects the high purity of the designed reaction.^25^ As Ni and Co hydroxides have similar structures, the differentiation of the two phases was not simple. The diffraction peaks around 11°, 23°, 33°, 34°, and 61° are attributed to the (003), (006), (220), (012), and (113) planes of α-Co(OH)_2_ and α-Ni(OH)_2_ (Joint Committee on Powder Diffraction Standards (JCPDS) 460605 and 380717, respectively). The 2*θ* diffraction peak at 11.6° (0.76 nm) corresponds to both α-Co(OH)_2_ and α-Ni(OH)_2_. The stacking of the layers along the *c*-axis indicates the difference between the α- and β-phases. The Co(OH)_2_ and Ni(OH)_2_ layers are randomly oriented and separated by intercalated water molecules bonded to the hydroxyl groups by hydrogen bonds in the α-phase with an interlamellar distance of approximately 0.8 nm. In the β-phase, Co(OH)_2_ and Ni(OH)_2_ layers are perfectly stacked along the *c*-axis with an interlamellar distance of 0.4 nm without intercalated species. α-phase compounds are theoretically expected to exhibit superior electrochemical activities to those of the β-phase compounds.^26^ The peak at 19.38° observed for the sample synthesized for 12 h corresponds to the (001) plane and confirms the layered structure of the synthesized NiCo hydroxide.

**Fig. 3:**
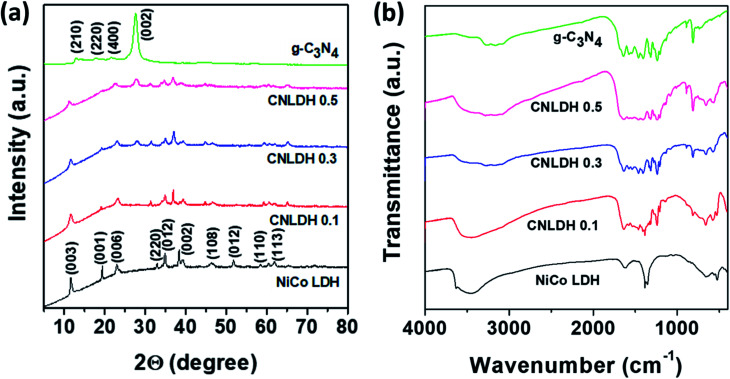
(a) XRD patterns and (b) FTIR spectra of g-C_3_N_4_, CNLDH 0.1, CNLDH 0.3, CNLDH 0.5, and NiCo LDH.

Fig. 3a shows the XRD patterns of the NiCo LDH, g-C_3_N_4_, and CNLDH nanohybrids. The peaks at 12.74° and 27.43° observed for g-C_3_N_4_ can be indexed to the (210) and (002) planes (JCPDS 000660813), corresponding to the in-plane structural packing of tri-*s*-triazine units with a distance of 0.68 nm and periodic interlayer stacking of aromatic segments with a distance of 0.32 nm in the conjugated aromatic system, respectively. The peaks corresponding to the (002) plane are usually attributed to the distance between the layers of the graphitic material; however, for g-C_3_N_4_, the peaks corresponding to the (210) plane are attributed to the intralayer *d*-spacing.^27^ In addition, the *d*-spacing of the (002) plane of CNLDH 0.1 suggests that g-C_3_N_4_ is intercalated between the LDH layers. Further, the high-angle (2*θ*) shifts of the (002) XRD peaks of the LDH composites (Fig. S3a†) indicate decreased stacking distances and reveal that the packing density of g-C_3_N_4_ and LDH perpendicular to the layer direction is higher. This is attributed mainly to the van der Waals interaction between two adjacent layers.^28^ The left shift of the (003) diffraction peak (Fig. S3b†) reflects the increased crystal lattice spacing of the LDH owing to the intercalation of g-C_3_N_4_, showing that the CNLDH structure is a hybrid consisting of two phases. In addition, as shown in Fig. S3a,† the intensity of the characteristic (002) diffraction peak of g-C_3_N_4_ increases with an increase in the content of g-C_3_N_4_ in the CNLDH hybrid, reflecting the intercalation of g-C_3_N_4_ in the NiCo LDH nanostructure, which increased the interlayer spacing.^29,30^

### FTIR spectroscopy

The functional groups in the prepared samples (NiCo LDH, g-C_3_N_4_, and CNLDH nanohybrids) are analyzed by FTIR spectroscopy (Fig. 3b). The narrow band around 3634 cm^−1^ in the FTIR spectrum of the NiCo LDH sample corresponding to the stretching vibration of OH^−^ groups reflects the replacement of the carboxyl organic ligand by OH^−^.^31^ The characteristic bands of the NiCo LDH at 3454 and 1630 cm^−1^ originate from the O–H stretching vibration and bending modes of the interlayer water and hydroxyl group, respectively. The band at 1383 cm^−1^ is assigned to the vibration of interlayer CO_3_^2−^ and NO_3_^−^ anions. CO_3_^2−^ participated in the formation of the nickel cobalt carbonate hydroxide hydrate with Ni^2+^ and Co^2+^ ions through coordinate bonds, while NO_3_^−^ was retained in the interlayer LDH.^32^ Furthermore, the additional absorption signals under 1000 cm^−1^ are attributed to the stretching and bending vibrations of Ni–O–H and Co–O–H in the LDH.^33^ The FTIR spectrum of g-C_3_N_4_ shows a broad absorption peak at 3000–3600 cm^−1^ attributed to the stretching vibrations of N–H groups. The typical stretching modes of CN heterocycles are observed in the range of 1200–1650 cm^−1^, while the absorption at 810 cm^−1^ is assigned to tri-*s*-triazine, *i.e.*, to the breathing mode of triazine.^34^ The FTIR spectra are in good agreement with the previous results on g-C_3_N_4_ materials prepared by polycondensation and polymerization reactions.^35,36^ Notably, with the increase in g-C_3_N_4_ content in the LDH, the stretching vibrations of the CN heterocycles are observed along with the breathing mode of triazine in the FTIR spectrum of the hybrid, which suggests the hybridization of g-C_3_N_4_ and ultrathin NiCo LDH nanosheets.^37^ The FTIR spectra reveal the CN structure on the surface of the LDH and strong electrostatic bonding interaction between g-C_3_N_4_ and LDH.

### XPS

**Fig. 4:**
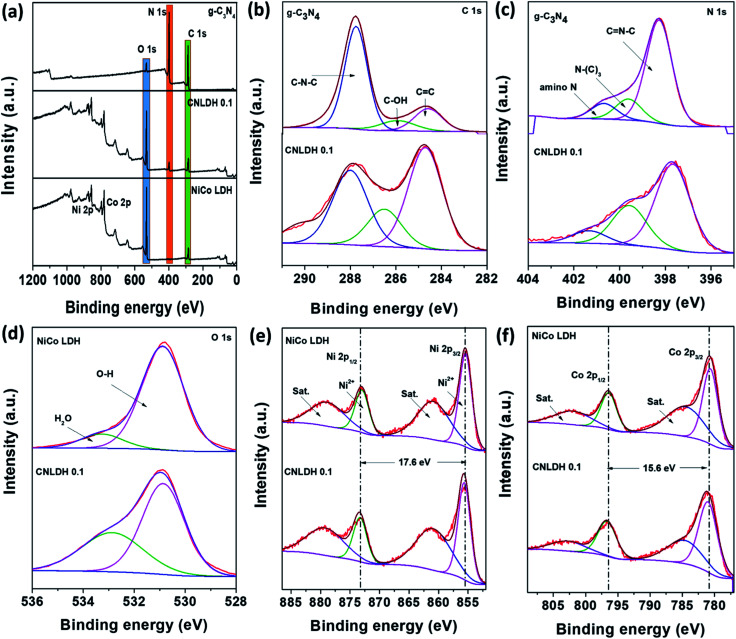
XP spectra of g-C_3_N_4_, NiCo LDH, and CNLDH 0.1.

The XP survey spectra of g-C_3_N_4_, NiCo LDH, and CNLDH 0.1 in Fig. 4a reveal the presence of C and N in CNLDH 0.1. The XP survey spectrum of g-C_3_N_4_ shows high C 1s and N 1s peaks and small O 1s peak corresponding to an atomic ratio of 42.8 : 55.7 : 1.5. The high-resolution C 1s spectrum in Fig. 4b can be fitted by Gaussian curves with dominant components centered at 287.7, 285.9, and 284.6 eV, attributed to C–N–C, C–OH, and sp^2^ C–C bonds of graphitic carbon, respectively. Similarly, the N 1s peak (Fig. 4c) can be deconvoluted into three peaks centered at 398.5, 399.6, and 400.6 eV, corresponding to C=N–C, tertiary nitrogen (N–(C_3_)), and amino functional group having a hydrogen atom, respectively. The synthesis of a defect-free g-C_3_N_4_ with the perfect stoichiometric ratio of 0.75 is often challenging. The obtained C/N ratio of 0.76 of our g-C_3_N_4_ is attributed to the various optimizations carried out along with the systematic reaction design (Table S1†).^38^ The C 1s and N 1s peaks of CNLDH 0.1 confirm the formation of the heterostructure. The binding energies of the C 1s and N 1s core electrons remain almost the same, suggesting similar chemical states of carbon and nitrogen (Fig. 4a). However, the percentage of sp^2^ C=C is higher in the hybrid than in g-C_3_N_4_ (shown in Fig. 4b), suggesting that tri-*s*-triazine units were connected with amino groups between the layers.^39^ The C–N–C and C–OH peak intensities of CNLDH 0.1 are reduced compared with those of g-C_3_N_4_ indicating the prominent contents of Ni and Co ions on C–N–C functional groups of CN. In addition, the positive binding energy shifts of the C=N–C, N–(C)_3_, and amino N peaks of CNLDH 0.1 confirm the intercalation between NiCo LDH and g-C_3_N_4_ sheets (Fig. 4c).^40^ The presence of elemental C in the NiCo LDH could be attributed to the residual carbon from the sample and adventitious hydrocarbons from the XPS instrument. The Ni 2p XP spectrum (Fig. 4e) shows 2p_3/2_ and 2p_1/2_ peaks at binding energies of 855.6 and 873.2 eV, respectively, separated by a binding energy of 17.6 eV, which reflects the formation of the NiCo hydroxide. The Co 2p high-resolution spectrum can be fitted into two doublet spin orbitals of 2p_3/2_ and 2p_1/2_ with two shakeup satellites. In addition, the deconvoluted O 1s spectrum shows two peaks at binding energies of 530.8 and 533.3 eV attributed to hydroxyl ions and OH^−^ of absorbed molecules, respectively.^41^ The XPS analysis demonstrated that the NiCo LDH nanosheets not only were attached physically on the surfaces of the g-C_3_N_4_ sheets, but also formed heterostructures with the g-C_3_N_4_ sheets.

### BET analysis

The as-fabricated g-C_3_N_4_, NiCo LDH, and CNLDH 0.1 (Fig. S4a, c, and e,† respectively) exhibit type-IV nitrogen adsorption–desorption isotherms with large hysteresis loops, indicating the mesoporous structures. The average pore size is reduced upon the addition of g-C_3_N_4_ to the NiCo LDH. This can be attributed to the incorporation of NiCo LDH particles between the pores of the lamellar g-C_3_N_4_. Remarkably, the BET specific surface area of CNLDH 0.1 is 69.0 m^2^ g^−1^, approximately three times higher than that of the pure NiCo LDH (24.2 m^2^ g^−1^), which indicates that the introduction of the small amount of g-C_3_N_4_ relieves the agglomeration of NiCo LDH layers. Fig. S4b, d, and f† present the pore size distributions of g-C_3_N_4_, NiCo LDH, and CNLDH 0.1, respectively, showing the average pore sizes of 5 nm.

### Electrochemical properties

**Fig. 5:**
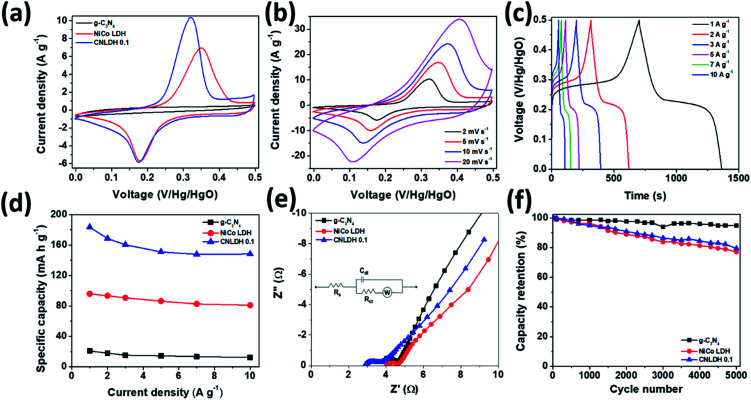
Electrochemical performances of g-C_3_N_4_, NiCo LDH, and CNLDH 0.1. (a) CV curves of g-C_3_N_4_, NiCo LDH, and CNLDH 0.1 at 2 mV s^−1^. (b) CV curves of CNLDH 0.1. (c) GCD curves of CNLDH 0.1 at different current densities. (d) Variation in specific capacity with the current density. (e) EIS and (f) electrochemical cyclic stability test results at 10 A g^−1^ in a 6 M KOH electrolyte for g-C_3_N_4_, NiCo LDH, and CNLDH 0.1. The inset (e) shows the equivalent circuit model used for fitting the EIS spectra.

The performance metrics of our newly synthesized g-C_3_N_4_, NiCo LDH, and CNLDH heterostructures were evaluated by electrochemical measurements in a three-electrode system. Fig. 5a shows the CV curves of g-C_3_N_4_, NiCo LDH, and CNLDH 0.1 at a scan rate of 2 mV s^−1^. The rectangular CV curves of g-C_3_N_4_ (Fig. S5a†) in the potential range of 0 to 0.5 V reveal its double-layer capacitive electrochemical behavior, whereas the NiCo LDH exhibits Faradaic redox peaks (Fig. S5c†). The area of the CV curve of CNLDH 0.1 is considerably larger than those of the other electrodes, owing to the higher capacity of CNLDH 0.1 than those of g-C_3_N_4_ and NiCo LDH. This is in agreement with the capacities estimated using the GCD curves (Fig. 5d). The CV curve area of the g-C_3_N_4_ electrode is very small compared to that of the NiCo LDH, suggesting a very small capacitive contribution of g-C_3_N_4_ in CNLDH 0.1. However, the resultant nanohybrid electrode exhibited a twofold increase in specific capacity compared to that of the NiCo LDH. This could be attributed to the large surface active area provided by g-C_3_N_4_ to effectively transfer electrons and restrict the stacking of the LDH. The CNLDH 0.1 electrode exhibited the maximum specific capacity of 183.43 mA h g^−1^ at a current density of 1 A g^−1^, higher than those of g-C_3_N_4_ and NiCo LDH of 20.89 mA h g^−1^ and 95.92 mA h g^−1^, respectively. Notably, CNLDH 0.1 exhibits the best specific capacity among those of the previously reported structures presented in Table 1.^42–47^ The series of optimizations shows that CNLDH 0.1 is superior to CNLDH 0.3 and CNLDH 0.5 (Fig. S6†). Therefore, we use CNLDH 0.1 as a representative sample to discuss the corresponding physical, chemical, and electrochemical properties. Further, the CV curves of the CNLDH 0.1 electrode (Fig. 5b) exhibit a distinct pair of redox peaks at 0.32 and 0.17 V *vs.* Hg/HgO, related to the electrochemical redox reactions of Ni(OH)_2_/NiOOH and CoOOH/CoO_2_ in the NiCo LDH, respectively.^48^ With the increase in scan rate, the anodic and cathodic peaks shift toward positive and negative potentials, respectively, owing to the polarization of the electrode.^49^ Charge–discharge curves of CNLDH 0.1 at different current densities are shown in Fig. 5c. CNLDH 0.1 exhibits the highest specific capacity indicating that the content of added g-C_3_N_4_ in the composite is most suitable for the full utilization of the layered LDH (Fig. 5d). The relation between *C*_sp_ and current density shows the higher capacity of CNLDH 0.1 than those of the g-C_3_N_4_ and NiCo LDH electrodes at each current density. Moreover, the *C*_sp_ values of all samples gradually decrease with the increase in current density. At a low current density, the electrolyte ions have sufficient time to diffuse and well contact the active surfaces for complete reaction, and thus full utilization of the active material.

**Table 1:** Electrochemical performances of CNLDH 0.1 and previously reported structures

	Electrode	Electrolyte	*C*_sp_	Reference
1	NiSe@MoSe_2_	2 M KOH	128.2 mA h g^−1^ at 1 A g^−1^	42
2	2D Ti_3_C_2_/Ni_2_CO_3_(OH)_2_	6 M KOH	173.8 mA h g^−1^ at 1 A g^−1^	43
3	CNT@Ni(OH)_2_	1 M KOH	167.0 mA h g^−1^ at 2 A g^−1^	44
4	NCO_*x*_/Er-GO	1 M KOH	180.6 mA h g^−1^ at 1 A g^−1^	45
5	Ni_0.28_Co_0.72_(OH)_2_	6 M KOH	174.3 mA h g^−1^ at 1 A g^−1^	46
6	N, S, codoped Ni_0.75_Co_0.25_(CO_3_)_0.125_(OH)_2_	1 M KOH	137.7 mA h g^−1^ at 0.5 A g^−1^	47
7	CNLDH 0.1	6 M KOH	182.43 mA h g^−1^ at 1 A g^−1^	This study

However, at a high current density, only the external active surface can be utilized for charge storage.^50^ The CNLDH 0.1 nanohybrid electrode retains approximately 76% of its initial capacity when the current density is increased from 1 to 10 A g^−1^, while that of the pure g-C_3_N_4_ decreases to 10%. The NiCo LDH has an excellent rate capability of 81%, which helps improve the rate capability of CNLDH 0.1. Fig. 5e shows the Nyquist plots of pure g-C_3_N_4_, NiCo LDH, and CNLDH 0.1. An equivalent circuit is used to fit the impedance spectrum (inset Fig. 5e), where *R*_s_ represents the solution resistance, *C*_dl_ is double layer capacitance, *R*_ct_ gives the value of charge transfer resistance, and *W* is Warburg impedance. The *R*_s_ and *R*_ct_ values were calculated by reading the real axis intercepts at high frequency region of the Nyquist plot. CNLDH 0.1 shows lower *R*_s_ (2.91 Ω) and *R*_ct_ (3.72 Ω) compared to pure g-C_3_N_4_ (*R*_s_ = 3.71 Ω and *R*_ct_ = 4.63 Ω) and NiCo LDH (*R*_s_ = 4.02 Ω and *R*_ct_ = 4.67 Ω). The superior electronic transport in CNLDH 0.1, attributed to the enhanced interfacial contact by the anchoring of g-C_3_N_4_ on the layered material (NiCo LDH). The improved interfacial contact promotes a fast and smooth ion diffusion through the layered structure and decreases its transfer resistance. To evaluate the application feasibility of these materials, the electrochemical cycling stabilities of g-C_3_N_4_, NiCo LDH, and CNLDH 0.1 were evaluated. Fig. 5f shows the capacitive stabilities of g-C_3_N_4_, NiCo LDH, and CNLDH 0.1 at a current density of 10 A g^−1^. CNLDH 0.1 retains 79% of its initial capacity after 5000 cycles showing its long-term stability. The corresponding GCD curves are shown in Fig. S7.† The increased electrochemical performances of the CNLDH 0.1 nanohybrid electrode are attributed to the synergetic effect of the g-C_3_N_4_ and NiCo LDH 2D nanostructures.

**Fig. 6:**
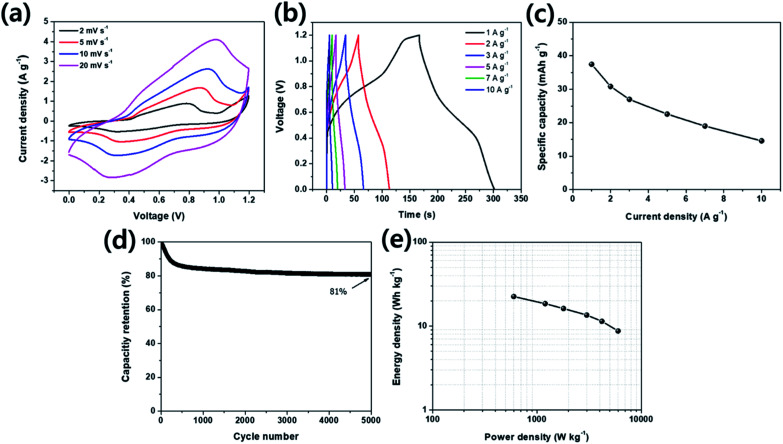
Electrochemical performance of the CNLDH 0.1//AC hybrid supercapacitor. (a) CV curves at various scan rates within a potential range of 0–1.2 V, (b) GCD curves and (c) corresponding specific capacity at various current densities. (d) Capacity retention at 10 A g^−1^ and (e) Ragone plot of CNLDH 0.1//AC hybrid supercapacitor.

For evaluating the practical application characteristics of CNLDH 0.1 electrode material, a hybrid supercapacitor using CNLDH 0.1 as a positive electrode and activated carbon (AC) as a negative electrode was assembled. Fig. 6 illustrates the electrochemical performance of CNLDH 0.1//AC hybrid supercapacitor in 6 M KOH electrolyte. From Fig. 6a, it can be seen that the hybrid device expands the working potential window up to 1.2 V. As the hybrid device is the combination of redox electrode and EDLC electrode, the CV curves show rectangular shapes with slightly redox peaks. Fig. 6b shows the GCD curves of CNLHD 0.1//AC hybrid supercapacitor at different current densities. The maximum specific capacity of 37.44 mA h g^−1^ is obtained at a current density of 1 A g^−1^. The specific capacity at current densities of 2, 3, 5, 7 and 10 A g^−1^ are 30.81, 26.96, 22.51, 18.96 and 14.55 mA h g^−1^, respectively (Fig. 6c). The capacity retention of hybrid supercapacitor is 81% after 5000 cycles at a current density of 10 A g^−1^, showing excellent electrochemical stability of the CNLDH 0.1//AC hybrid supercapacitor. The Ragone plot (Fig. 6e) of CNLDH 0.1//AC hybrid supercapacitor shows the maximum energy density of 22.46 W h kg^−1^ at a power density of 600 W kg^−1^. The obtained values of energy density and power density of CNLDH 0.1//AC hybrid supercapacitor are quite remarkable compared to reported hybrid supercapacitor assembly.^51–54^ These electrochemical analyses of CNLDH 0.1//AC hybrid supercapacitor confirm that CNLDH 0.1 nanohybrid is an impressive electrode material for supercapacitor application.

## Conclusion

We presented a simple strategy for the development of CNLDH nanohybrids of g-C_3_N_4_ and 2D NiCo layered materials for supercapacitor electrodes. A series of CNLDH nanohybrids with different mass ratios of g-C_3_N_4_ and NiCo LDH was prepared by the hydrothermal method. The structures and morphologies of the nanohybrids suggested that the NiCo LDH nanosheets were *in situ*-anchored on the surfaces of the g-C_3_N_4_ nanosheets or *vice versa*. CNLDH 0.1 exhibited excellent electrochemical performances, higher than those of CNLDH 0.3 and CNLDH 0.5, owing to the synergetic effect of g-C_3_N_4_ and NiCo LDH. In addition, CNLDH 0.1 exhibited a high specific capacity (182.43 mA h g^−1^ at 1 A g^−1^) and long-term cycling stability. The assembled CNLDH 0.1//AC hybrid supercapacitor presented the maximum energy density of 22.46 W h kg^−1^ at a power density of 600 W kg^−1^ and maintained an energy density of 8.73 W h kg^−1^ at a high power density of 6000 W kg^−1^. These results indicate that the CNLDH nanohybrid is a promising material for use as a supercapacitor electrode.

## Conflicts of interest

There are no conflicts to declare.

## Supplementary Material

RA-009-C9RA06068E-s001

## References

[cit1] Sun Y. Q., Wu Q. O., Shi G. Q. (2011). Energy Environ. Sci..

[cit2] Wu X., Zeng Y., Gao H., Su J., Liu J., Zhu Z. (2013). J. Mater. Chem. A.

[cit3] Zhao C., Ju P., Wang S., Zhang Y., Min S., Qian X. (2016). Electrochim. Acta.

[cit4] Lee D. U., Fu J., Park M. G., Liu H., Kashkooli A. G., Chen Z. (2016). Nano Lett..

[cit5] Liu J., Wang J., Xu C., Jiang H., Li C., Zhang L., Lin J., Shen Z. X. (2018). Adv. Sci..

[cit6] Wilson J. A., Yoffe A. D. (1969). Adv. Phys..

[cit7] Mas-Ballesté R., Gómez-Navarro C., Gómez-Herrero J., Zamora F. (2011). Nanoscale.

[cit8] Miró P., Audiffred M., Heine T. (2014). Chem. Soc. Rev..

[cit9] Geim A. K., Grigorieva I. V. (2013). Nature.

[cit10] Novoselov K. S., Mishchenko A., Carvalho A., Castro Neto A. H. (2016). Science.

[cit11] Pomerantseva E., Gogotsi Y. (2017). Nat. Energy.

[cit12] Irshad R., Tahir K., Li B., Sher Z., Ali J., Nazir S. (2018). J. Ind. Eng. Chem..

[cit13] Han S. A., Sohn A., Kim S. W. (2017). FlatChem.

[cit14] Zhao R., Gao J., Mei S., Wu Y., Wang X., Zhai X., Yang J., Hao C., Yan J. (2017). Nanotechnology.

[cit15] Finge T., Riederer F., Mueller M. R., Grap T., Kallis K., Knoch J. (2017). Ann. Phys..

[cit16] Yi F., Ren H., Shan J., Sun X., Wei D., Liu Z. (2018). Chem. Soc. Rev..

[cit17] Tian N., Zhang Y., Li X., Xiao K., Du X., Dong F., Waterhouse G. I. N., Zhang T., Huang H. (2017). Nano Energy.

[cit18] Peng L., Fang Z., Zhu Y., Yan C., Yu G. (2018). Adv. Energy Mater..

[cit19] Li X., Du D., Zhang Y., Xing W., Xue Q., Yan Z. (2017). J. Mater. Chem. A.

[cit20] Yang Y., Chen J., Mao Z., An N., Wang D., Fahlman B. D. (2017). RSC Adv..

[cit21] Ong W. J., Tan L. L., Ng Y. H., Yong S. T., Chai S. P. (2016). Chem. Rev..

[cit22] Wu Z., Li L., Yan J., Zhang X. (2017). Adv. Sci..

[cit23] Fang J., Li M., Li Q., Zhang W., Shou Q., Liu F., Zhang X., Cheng J. (2012). Electrochim. Acta.

[cit24] Yang J., Zhu J., Xu J., Zhang C., Liu T. (2017). ACS Appl. Mater. Interfaces.

[cit25] Guellati O., Harat A., Momodu D., Dangbegnon J., Romero T., Begin D., Pham-Huu C., Manyala N., Guerioune M. (2018). Electrochim. Acta.

[cit26] Hu Z., Xie Y. L., Wang Y. X., Wu H. Y., Yang Y. Y., Zhang Z. Y. (2009). Electrochim. Acta.

[cit27] Fina F., Callear S. K., Carins G. M., Irvine J. T. S. (2015). Chem. Mater..

[cit28] Dai H., Gao X., Liu E., Yang Y. H., Hou W. Q., Kang L. M., Fan J., Hu X. (2013). Diamond Relat. Mater..

[cit29] Yu J., Wang S., Cheng B., Lin Z., Huang F. (2013). Catal. Sci. Technol..

[cit30] Li H., Musharavati F., Zalenezhad E., Chen X., Hui K. N., Hui K. S. (2018). Electrochim. Acta.

[cit31] Nagaraju G., Raju G. S. R., Ko Y. H., Yu J. S. (2016). Nanoscale.

[cit32] Guan T., Fang L., Lu Y., Wu F., Ling F., Gao J., Hud B., Meng F., Jin X. (2017). Colloids Surf., A.

[cit33] Cao F., Gan M., Ma L., Li X., Yan F., Ye M., Zhai Y., Zhou Y. (2017). Synth. Met..

[cit34] Xu J., Zhang L., Shi R., Zhu Y. (2013). J. Mater. Chem. A.

[cit35] Zou H., Yan X., Ren J., Wu X., Dai Y., Sha D., Pan J., Liu J. (2015). J Materiomics..

[cit36] Xu M., Han L., Dong S. (2013). ACS Appl. Mater. Interfaces.

[cit37] Hu S. W., Yang L. W., Tian Y., Wei X. L., Ding J. W., Zhong J. X., Chu P. K. (2014). J. Colloid Interface Sci..

[cit38] Lakhi K. S., Park D. H., Bahily K. A., Cha W., Viswanathan B., Choyd J. H., Vinu A. (2017). Chem. Soc. Rev..

[cit39] Qiu P., Chen H., Xu C., Zhou N., Jiang F., Wang X., Fu Y. (2015). J. Mater. Chem. A.

[cit40] Wang T., Zhang S., Yan X., Lyu M., Wang L., Bell J., Wang H. (2017). ACS Appl. Mater. Interfaces.

[cit41] Guan T., Fang L., Lu Y., Wu F., Ling F., Gao J., Hu B., Meng F., Jin X. (2017). Colloids Surf., A.

[cit42] Peng J. H., Zhou J., Sun K., Ma G., Zhang Z., Feng E., Lei Z. (2017). ACS Sustainable Chem. Eng..

[cit43] Guo J., Zhao Y., Jiang N., Liu A., Gao L., Li Y., Wang H., Ma T. (2018). Electrochim. Acta.

[cit44] Yi H., Wang H. W., Jing Y. T., Peng T. Q., Wang Y. R., Guo J., He Q. L., Guo Z. H., Wang X. F. (2015). J. Mater. Chem. A.

[cit45] Adán-Mása A., Silvaa T. M., Guerlou-Demourguesb L., Bourgeoise L., Labrugere-Sarrosteg C., Montemora M. F. (2019). J. Power Sources.

[cit46] Tang Y., Liu Y., Yu S., Guo W., Mub S., Wang H., Zhao Y., Hou L., Fan Y., Gao F. (2015). Electrochim. Acta.

[cit47] Wen F., Zhang Y., Qian X., Zhang J., Hu R., Hu X., Wang X., Zhu J. (2017). ACS Appl. Mater. Interfaces.

[cit48] Zhou Q., Wang X., Liu Y., He Y., Gao Y., Liu J. (2014). J. Electrochem. Soc..

[cit49] Vogel Y. B., Zhang L., Darwish N., Gonçales V. R., Brun A. L., Gooding J. J., Molina A., Wallace G. G., Coote M. L., Gonzalez J., Ciamp S. (2017). Nat. Commun..

[cit50] Zhang J., Yang W., Liu J. (2016). Electrochim. Acta.

[cit51] Wang X., Sumboja A., Lin M. F., Yan J., Lee P. S. (2012). Nanoscale.

[cit52] Yu X., Lu B., Xu Z. (2014). Adv. Mater..

[cit53] Senthilkumar B., Meyrick D., Lee Y. S., Selvan R. K. (2013). RSC Adv..

[cit54] Jagadale A. D., Guan G., Li X., Du X., Ma X., Hao X., Abudula A. (2016). J. Power Sources.

