# Supplementary material for: Scalable nanohybrids of graphitic carbon nitride and layered NiCo hydroxide for high supercapacitive performance
Source: RSC Adv. 2019 Oct 18;9(58):33643–52. doi: 10.1039/c9ra06068e (PMC9073531; doi:10.1039/c9ra06068e)
Supplement: RA-009-C9RA06068E-s001 [file RA-009-C9RA06068E-s001.pdf]

Electronic Supplementary Material (ESI) for RSC Advances.
This journal is © The Royal Society of Chemistry 2019

Scalable nanohybrids of graphitic carbon nitride and layered NiCo hydroxide for high supercapacitive performance

Bebi Patil [a], Changyong Park [b], and Heejoon Ahn [a,b] *

[a]Institute of Nano Science and Technology, Hanyang University, Seoul 04763, South Korea

[b]Department of Organic and Nano Engineering, Hanyang University, Seoul 04763, South Korea

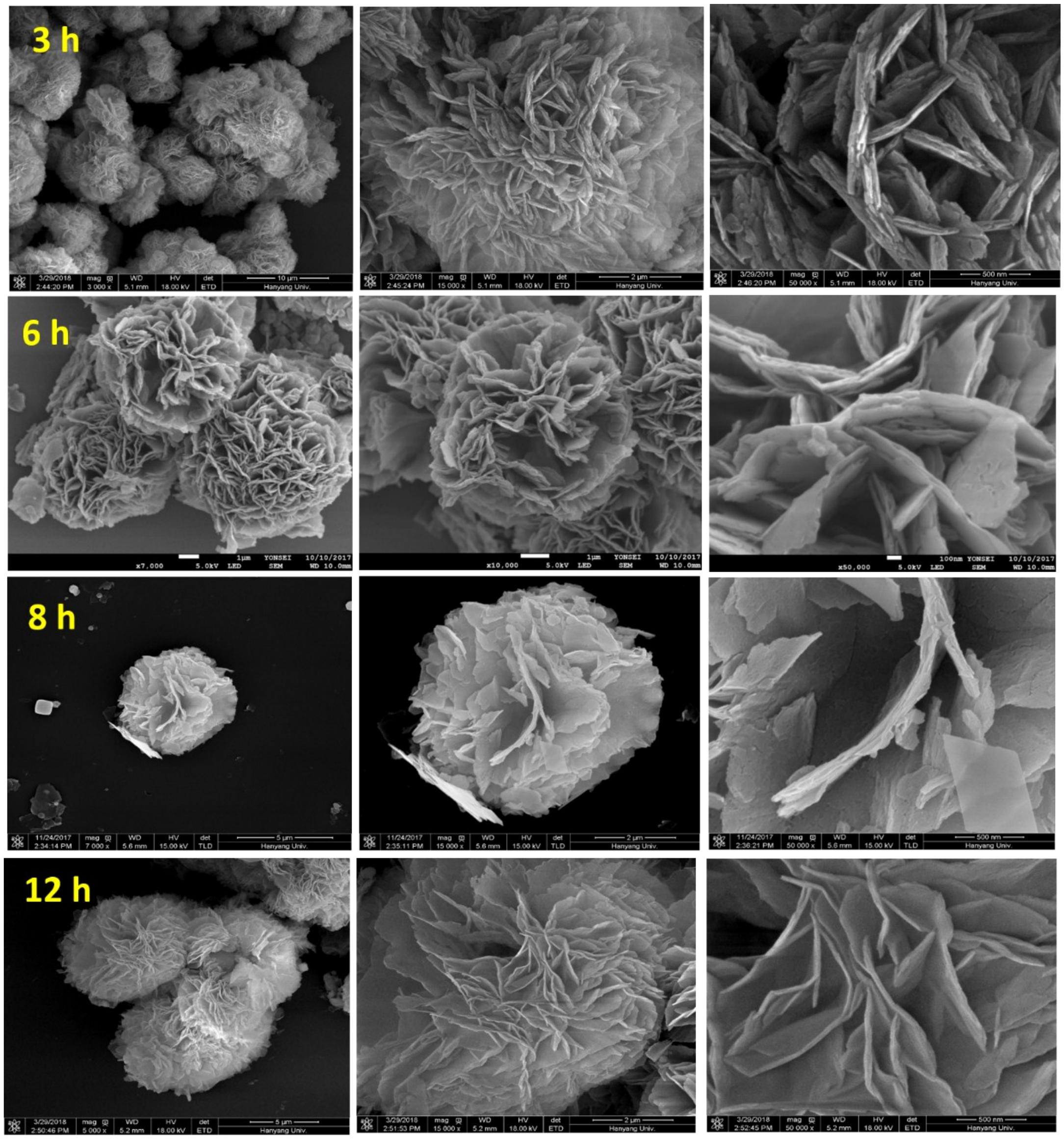

Fig. S1 SEM images of the NiCo LDHs obtained using different synthesis times.

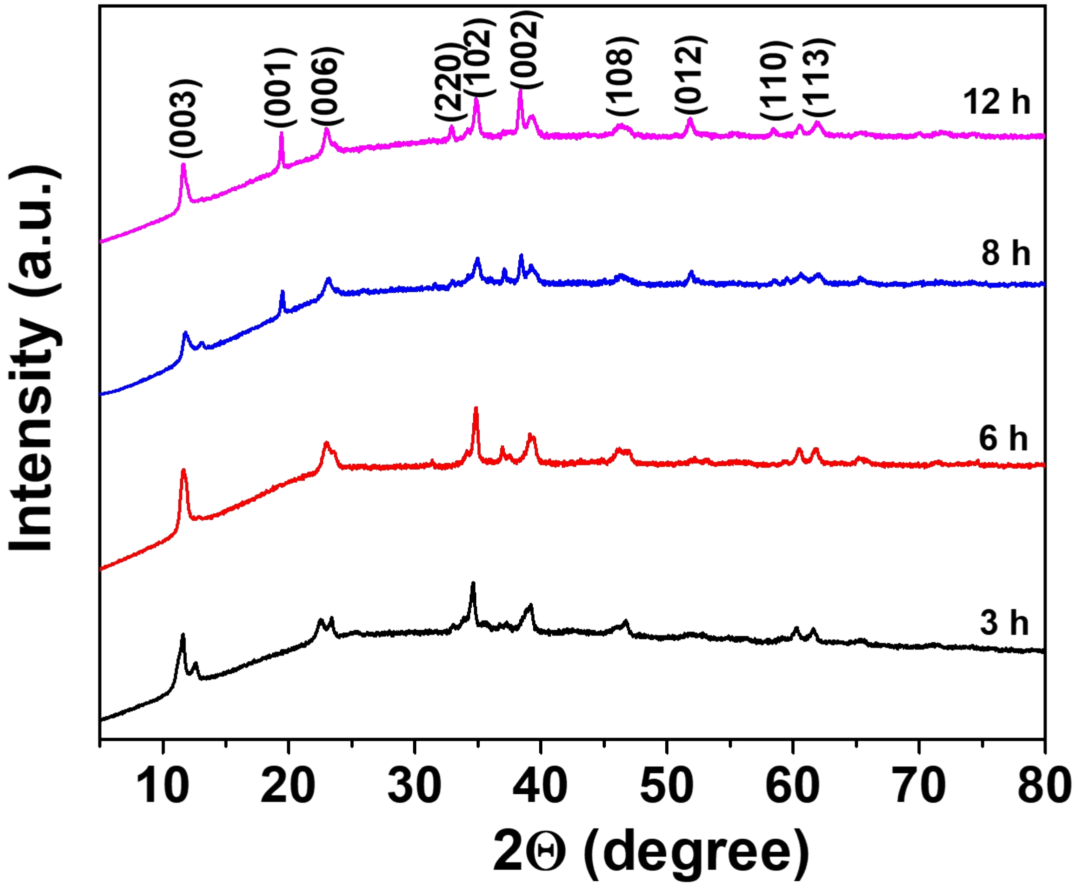

Fig. S2 XRD patterns of the NiCo LDHs synthesized for 3, 6, 8, and 12 h.

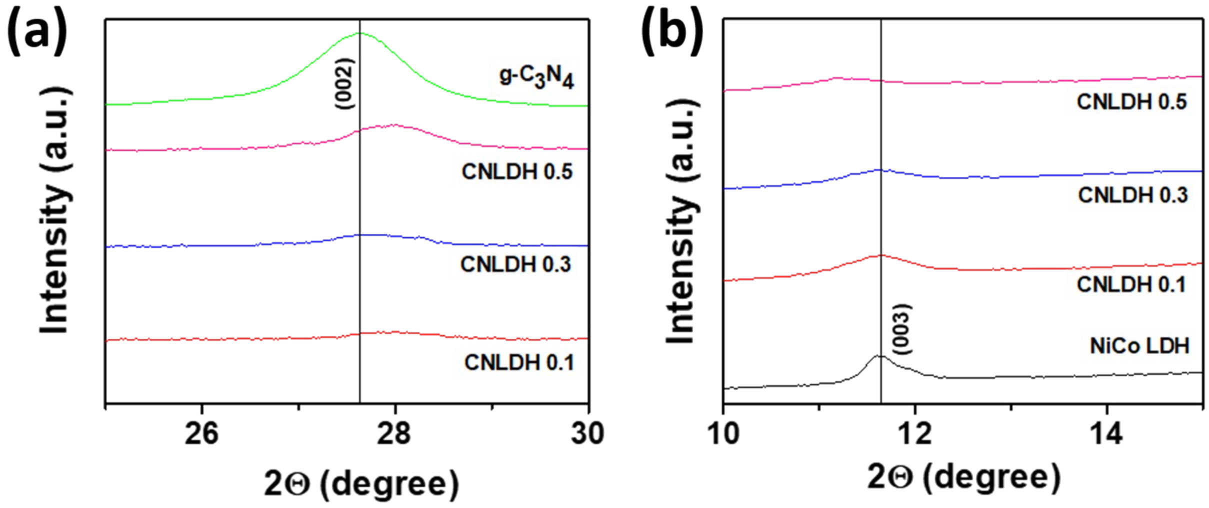

Fig. S3 XRD patterns showing the shifts of the (a) (002) and (b) (003) peaks.

Table S1. The elemental composition of C, N, O, Ni, and Co in g-C_3N_4, NiCo LDH and CNLDH 0.1 using XPS analysis.

	C (atomic %)	N (atomic %)	O (atomic %)	Ni (atomic %)	Co (atomic %)
g-C_3N_4	42.8	55.7	1.5	-	-
NiCo LDH	23.9	-	53	13.0	10.1
CNLDH 0.1	30.0	9.1	42.5	10.5	7.9

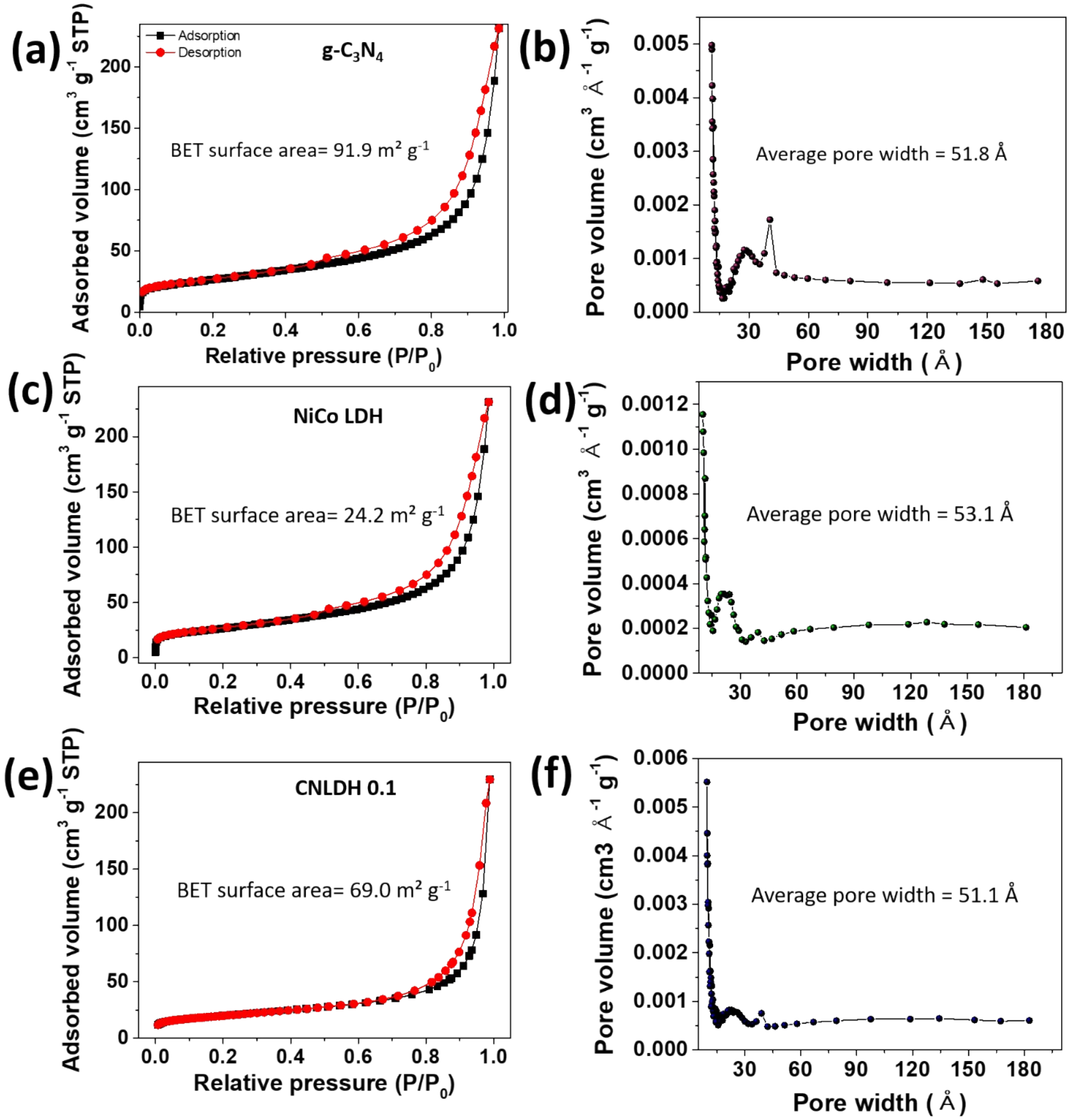

Fig. S4 Nitrogen adsorption–desorption isotherms and BJH pore size distributions of (a,b) g-C_3N_4, (c,d) NiCo LDH, and (e,f) CNLDH 0.1, respectively.

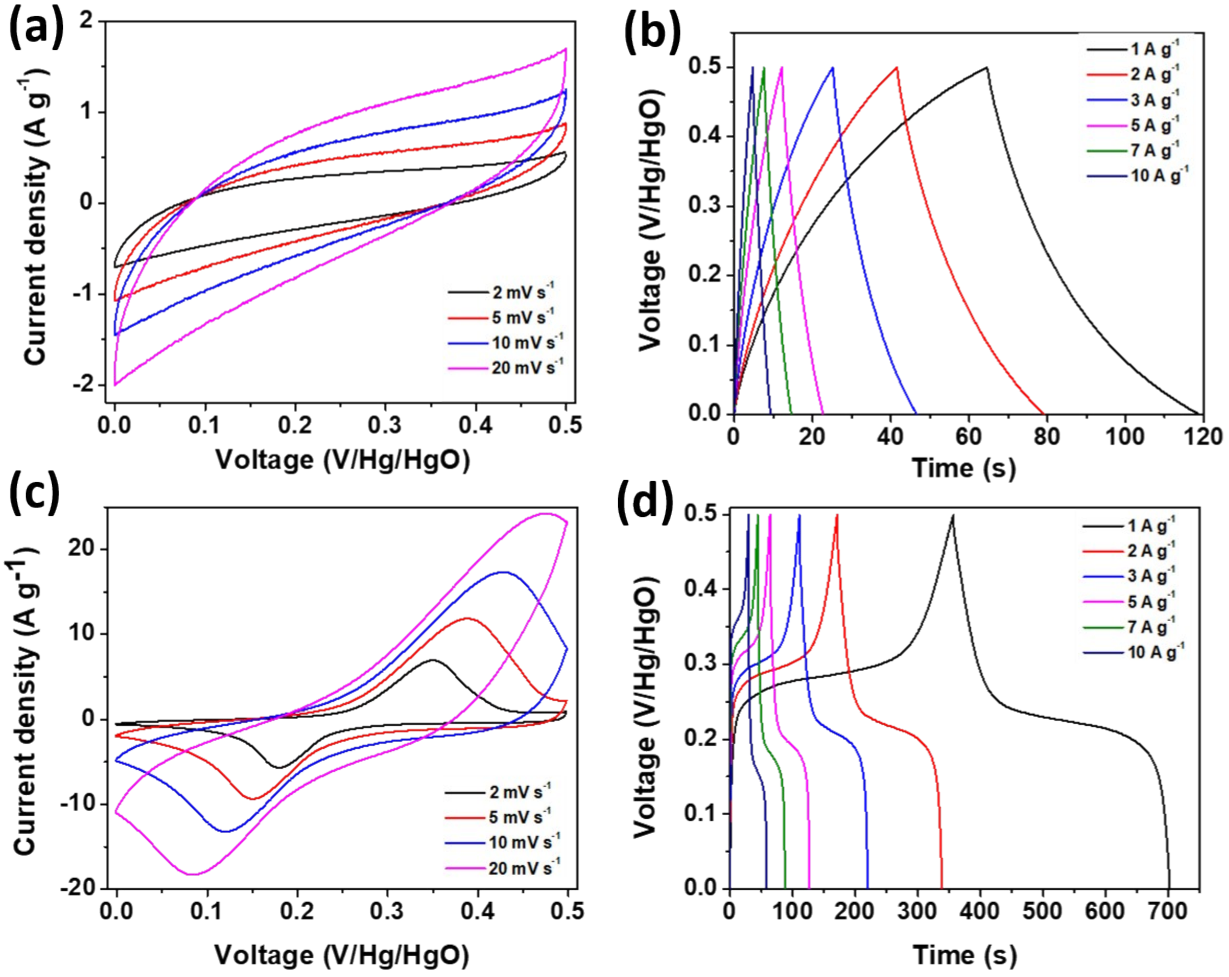

Fig. S5 CV and GCD curves of (a,b) g-C_3N_4 and (c,d) NiCo LDH at various scan rates and current densities, respectively.

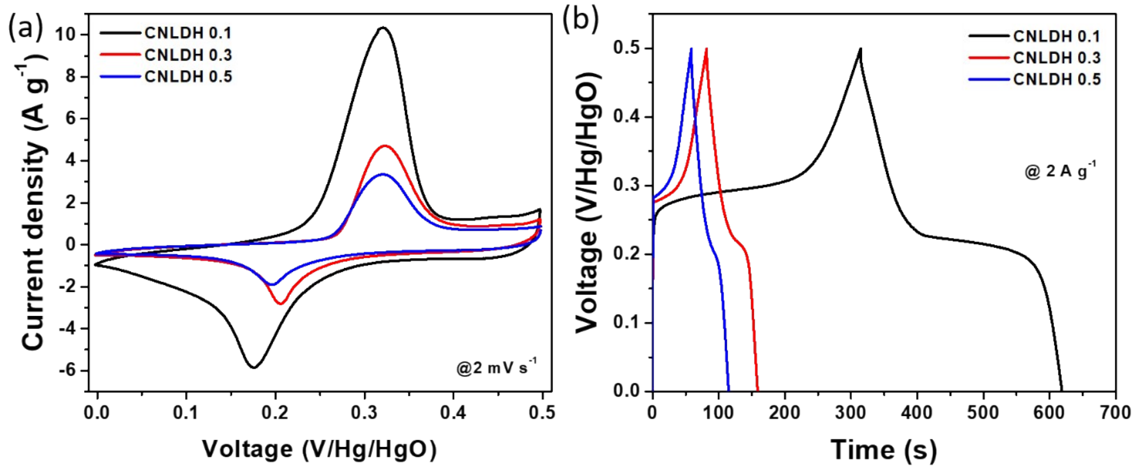

Fig. S6 (a) CV and (b) GCD curves of CNLDH 0.1, 0.3, and 0.5.

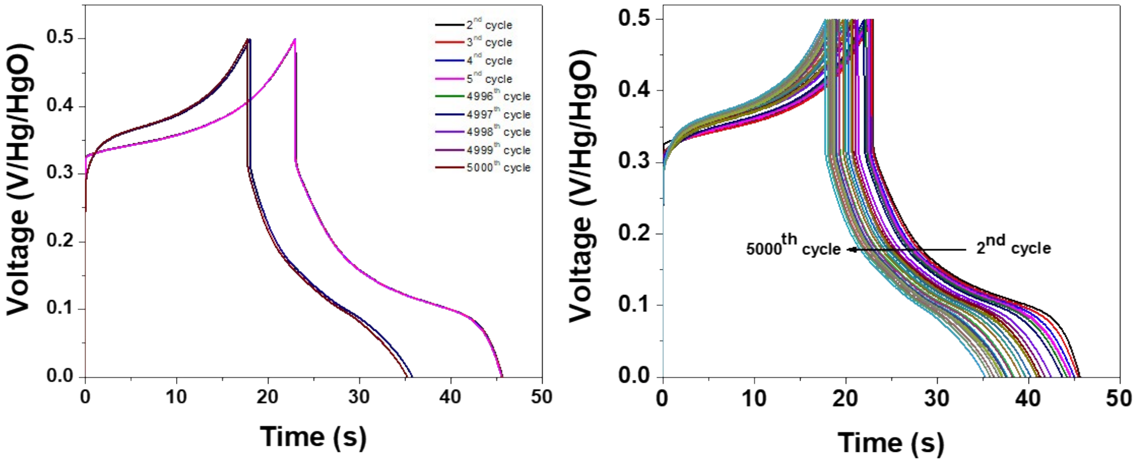

Fig. S7 Electrochemical cycling stability of CNLDH 0.1 electrode in 6 M KOH electrolyte.
